# Vital real-world experience regarding Naoshuantong capsules for unselected ischemic stroke (VENUS): Rationale, design, and baseline of a prospective, multicenter, observational study

**DOI:** 10.3389/fphar.2022.933258

**Published:** 2022-10-05

**Authors:** Xinglu Dong, Luda Feng, Tingting Li, Yuebo Song, Lei Li, Shengxian Wu, Chi Zhang, Ying Gao, ; For the VENUS Investigators

**Affiliations:** ^1^ Department of Neurology, Dongzhimen Hospital, Beijing University of Chinese Medicine, Beijing, China; ^2^ Institute for Brain Disorders, Beijing University of Chinese Medicine, Beijing, China; ^3^ Beijing University of Chinese Medicine, Beijing, China; ^4^ Chinese-Western Medicine Research and Development Working Committee, China Association of Traditional Chinese Medicine, Beijing, China; ^5^ Office of State Drug Clinical Trial Institution, Dongzhimen Hospital, Beijing University of Chinese Medicine, Beijing, China; ^6^ Dongzhimen Hospital, Beijing University of Chinese Medicine, Beijing, China

**Keywords:** stroke registry, ischemic stroke, Naoshuantong capsules, real-world setting, effectiveness, safety profile

## Abstract

**Background:** Naoshuantong capsules (NC) are commonly used for the treatment of ischemic stroke. Experimental research and small-sample clinical trials have demonstrated that NC is effective in improving neurological recovery. Yet, there is a substantial lack of high-quality evidence on the precision treatment population of NC and long-term safety when making real-world clinical decisions. The acquisition of prospective longitudinal data in the real-world setting is essential to fully characterize the effectiveness and safety profile of NC for patients with ischemic stroke.

**Methods:** The Vital real-world Experience regarding Naoshuantong capsules for Unselected ischemic Stroke (VENUS) registry is a prospective, multicenter, observational study aiming to register 5,000 patients. Eligible adult patients diagnosed with ischemic stroke and newly treated with NC within 30 days of symptom onset will be consecutively registered from 84 participating sites across the Chinese mainland. Baseline data will be recorded at the patient registry, and all patients will be regularly followed up at 2, 4, 8, and 12 weeks after the initial patient registry, and 180 days after ischemic stroke onset. The primary outcome is the distribution of scores on the modified Rankin Scale at 12 weeks after initial patient registry. Adverse events will be recorded during the study for NC safety assessment.

**Results:** A total of 4,185 patients with ischemic stroke were enrolled, among which 37.06% patients were female. The average age of all patients was 65.22 years. The proportion of patients whose course of ischemic stroke was less than 14 days accounted for 93.45%.

**Conclusion:** The VENUS registry is designed to comprehensively document medical data regarding NC treatment for ischemic stroke in real-world settings. The findings of this study will provide valuable insights into the clinical management of patients with ischemic stroke and the subsequent outcomes of the use of NC when included in the best clinical practice.

Study registration: This study was registered with the Chinese Clinical Trial Registry (URL: http://www.chictr.org.cn/index.aspx, Unique identifier: ChiCTR1900025053).

## Introduction

Stroke is the second leading cause of death worldwide and the first leading cause of death in China, where the mortality is twice that of the global average (Zhou et al., 2019; GBD 2019 Stroke Collaborators, 2021). Stroke leads to disabilities, and the recurrence of stroke must not be ignored (Coull et al., 2004; Chen et al., 2020). Stroke-related treatments and post-stroke care result in heavy economic burden. China has been reported to bear the highest economic burden related to stroke (Wang et al., 2017; Rajsic et al., 2019). The estimated lifelong risk of stroke is approximately 39.3% in China (GBD 2016 Lifetime Risk of Stroke Collaborators, 2018). Ischemic stroke (IS) is the main subtype of stroke accounting for 70–83% of all cases in China according to different survey data (Wang et al., 2017; Gu et al., 2021).

Although evidence-based interventions have been advocated in China, unavoidable gaps between guideline recommendations and clinical practice remain. These gaps may be attributed to the influence and challenge of known or unknown confounding factors or objective difficulties in real-world settings. Reperfusion treatment such as intravenous thrombolysis and endovascular treatment has been proven to improve functional outcomes of patients with acute ischemic stroke, but only a minority of highly selected patients could benefit from these treatments (Powers et al., 2019). Antiplatelet, lipid-lowering, and antihypertensive therapies make up the cornerstone of secondary prevention of IS, though irreversible aspirin or clopidogrel resistance of individuals and poor long-term medication compliance weaken the net benefit of recommended medicines (Wei et al., 2010; Wang et al., 2016; Girot et al., 2020). Therefore, there is an unmet need to investigate safe and effective therapies for IS when the optimal treatments are ineligible.

Naoshuantong capsules (NC) are a combination of five natural herbal ingredients that are widely used for the treatment of IS in China. Preclinical studies regarding their mechanisms have demonstrated that NC reduces the cerebral infarct area and attenuates neurological deficits *via* inhibiting neuronal apoptosis, suppressing the overexpression of inflammatory cascade injuries, attenuating the neurotoxicity of excitatory amino acids, and increasing hemorheology and cerebral energy metabolism (Xiang et al., 2010; Liu et al., 2014; Luo et al., 2020). These results have indicated that the efficacy of NC is based on a synergistic effect of several components, targets, and pathways. A systematic review of randomized controlled trials (RCTs) regarding the clinical efficacy and safety of NC reported benefits in alleviating neurological impairments and improving activities of daily living with 1–12 months of treatment in patients with IS.

However, unaddressed issues concerning NC treatment for IS in real-world practice limit its optimal usage. The epidemiology and clinical characteristics of patients with IS who undergo NC treatment remain unclear. RCTs that have reported the efficacy of NC included highly selected patients and excluded special populations such as elderly patients or patients with severe comorbidities, resulting in little data regarding the effects of NC in these populations. Additionally, the benefits and harms of NC for patients with IS should be interpreted with caution, as the RCTs were conducted under relatively ideal conditions without confounding factors, and a high risk of bias exists, challenging the robustness of the results regarding the efficacy and safety of NC (Zhang et al., 2019). The long-term effects of NC, such as the effects on functional status or stroke recurrence in patients with IS, remain uncertain due to a lack of data. The optimal time point for initiating NC treatment, proper dosage, and treatment course are yet to be verified. Although encouraging data have indicated the clinical safety of NC, the long-term safety in large-scale populations has not been investigated.

A prospective, multicenter registry with regular and long-term follow-up will provide the much-needed data regarding the use of NC in patients with IS. Therefore, the Vital real-world Experience regarding Naoshuantong capsules for Unselected ischemic Stroke (VENUS) registry has been developed to generate sociodemographic and medical data related to patients with IS who undergo NC treatment and to evaluate the effectiveness and safety of NC for patients with IS in a real-world setting.

## Methods and design

### Study design

**FIGURE 1:**
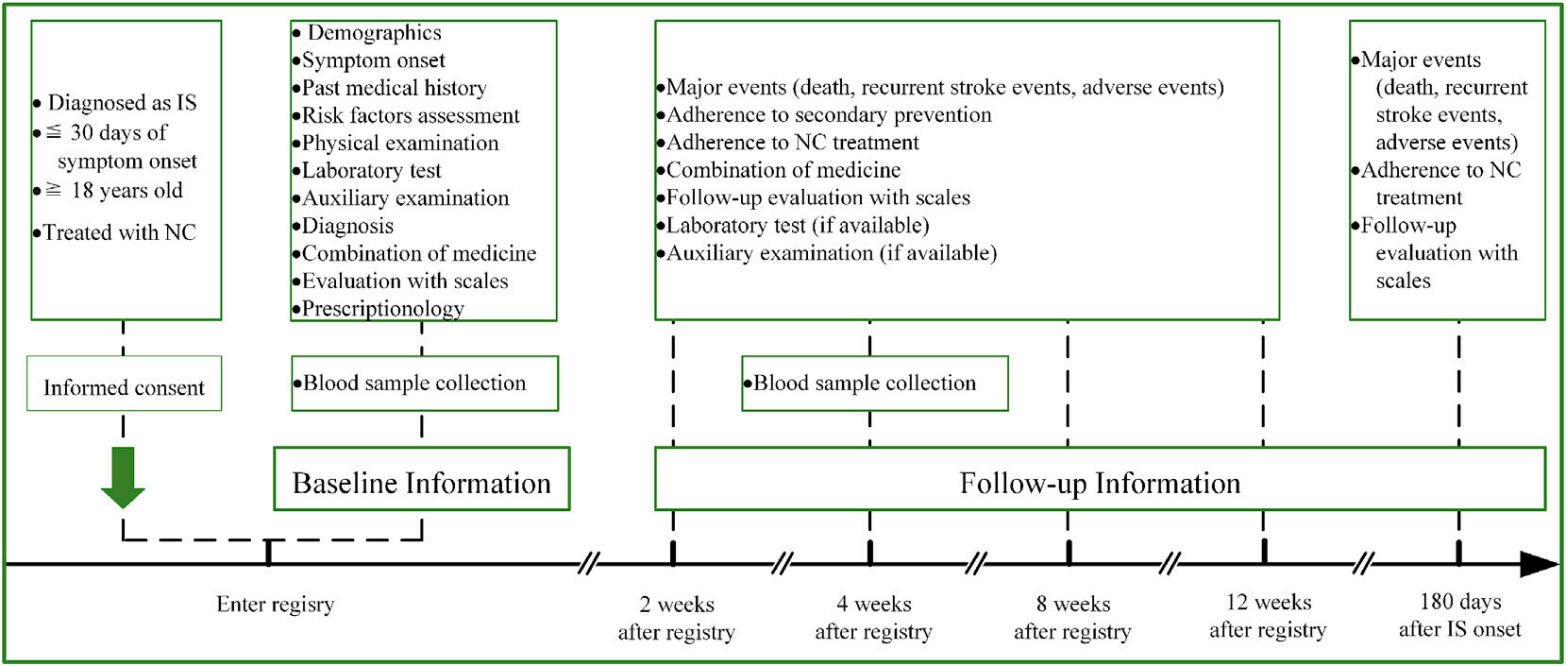
Flowchart of the VENUS registry. Abbreviations: IS, ischemic stroke; NC, Naoshuantong capsules.

The VENUS registry (registered with the Chinese Clinical Trial Registry, unique identifier: ChiCTR1900025053) is a prospective, multicenter, post-marketing, observational study designed to provide insight into the administration of NC for patients with IS in the real-world setting. The specific aims of the VENUS registry are to 1) systematically investigate the effectiveness of NC for patients with IS and explore any effectiveness difference among NC user cohorts with distinct clinical characteristics; 2) to observe the long-term safety profile of NC and probe potential risk factors *via* regular continuous follow-ups; 3) to establish a database for further nested studies with specific predefined hypotheses to facilitate novel knowledge discovery. An overview of the VENUS registry flowchart is shown in Figure 1. This study was approved by the institutional review board of the leading center, Dongzhimen Hospital, Beijing University of Chinese Medicine, Beijing, China (No. DZMEC-KY-2019-10), and was approved by the local institutional review boards of all participating sites.

### Study population

In order to achieve a high population representation, we registered patients with a unstrict inclusion criterion. All adult patients (≥18 years) admitted to the participating sites with a clear diagnosis of IS according to the guideline were screened. Patients who were newly treated with NC within 30 days of symptom onset were consecutively included in this study. Patients with other complications that hinder the assessment of neurological function and disability will be excluded. Patient participation is entirely voluntary and written informed consent that complies with the Declaration of Helsinki principle will be obtained from all patients or their legally authorized representatives.

### Specific treatment

Each 400-mg capsule of NC contains five herbal ingredients [*Typha angustifolia* L. (Pu Huang), *Paeonia lactiflora* Pall. (Chi Shao), *Paeonia lactiflora* Pall. (Yu Jin), *Gastrodia elata* Blume (Tian Ma), and *Leuzea uniflora* (L.) Holub (Lou Lu)] (see Appendix 1 and Appendix 2 in Supplementary Material). NC can be administered orally, or the contents can be diluted in water and administered *via* a gastric tube. The recommended dosage of NC is three capsules thrice per day, and the recommended treatment course is 4 weeks, but the actual dosage and treatment course of NC will be determined by the clinical physician according to the patients’ condition. All other Chinese medicines prescribed by the physicians will be truthfully recorded. Additionally, all patients will receive routine treatments according to the guidelines in terms of the early management and secondary prevention of IS (Powers et al., 2019; Kleindorfer et al., 2021), such as reperfusion treatment, antiplatelet, lipid lowering, antihypertension, and controlling of risk factors. Physicians will combine their clinical experiences and patients’ desires to make decisions on specific treatments when evidence-based therapies were ineligible.

### Data collection and management

The research personnel who are appointed at each participating site will be responsible for data collection. They will consecutively screen eligible patients and then record data using paper-based registry forms (PRF). Baseline data including patients’ demographics, past medical history, physical examination, laboratory test and auxiliary examination results, risk factor assessment, and the medical combination will be abstracted from the medical records. The symptom classification of IS will be judged based on the Oxfordshire Community Stroke Project (OCSP) criteria, and the etiological classification will be determined according to the Trial of Org 10172 in Acute Stroke Treatment (TOAST) criteria (Bamford et al., 1991; Adams et al., 1993). At baseline, the life dependency will be measured with the modified Rankin Scale (mRS), the neurological impairment will be measured with the National Institute of Health Stroke Scale (NIHSS), the activity of daily living will be measured with the Barthel Index (BI), the cognitive function will be measured with the Mini-Mental State Examination (MMSE), and the Chinese medicine syndrome will be measured with the Diagnostic Scale of Syndrome Elements in Ischemic Stroke (DSSEIS) (Folstein et al., 1975; Kasner, 2006; Gao et al., 2011; Gao et al., 2012). Additionally, the NC prescription details (dosage and frequency) and clinical physicians’ prescriptionology information will also be documented. The blood samples will be collected from 40 patients eligible from Dongzhimen Hospital and Xuanwu Hospital, Beijing, China. An independent data safety and monitoring board will conduct data monitoring to ensure adherence to study documentation, reporting procedures, and the study protocol.

### Follow-up procedures

The research personnel will regularly perform face-to-face or telephone follow-ups at 2, 4, 8, and 12 weeks after the initial patient registry, and 180 days after IS onset. Information including death, recurrent stroke events, and adverse events will be obtained during the whole study. Details of the follow-up timetable on life dependency, neurological impairment, activities of daily living, cognitive functions, and Chinese medicine syndrome collection are as follows: 1) assessment of mRS will be conducted at all follow-up time points mentioned previously; 2) assessment of DSSEIS, NIHSS, and MMSE will be conducted at 2, 4, 8, and 12 weeks after patient registry; 3) assessment of BI will be conducted at 2, 4, and 8 weeks after patient registry. The aforementioned blood samples will be collected again from 40 patients, at 4 weeks after patient registry. Adherence to the NC treatment and medicine combination will be recorded at 2, 4, 8, and 12 weeks after patient registry. The results will be recorded if the patients receive examination with regard to laboratory tests, magnetic resonance imaging (MRI) or computed tomography (CT), and transcranial Doppler sonography. Death will be confirmed by a death certificate. Recurrent stroke events leading to rehospitalization will be confirmed by discharge diagnosis while suspected recurrent stroke events will be determined by the endpoint judgment committee when without hospitalization (Feng et al., 2021). Adherence to the NC treatment will be defined as the proportion of prescribed doses taken (Osterberg and Blaschke, 2005).

### Study outcomes

The primary outcomes of the VENUS registry is the changes of life dependency measured with the distribution of scores on the mRS at 12 weeks after initial patient registry. The secondary outcomes are measured with different indicators those will be assessed at subsequent follow-up visits, such as changes in neurological impairment, long-term safety, improvements in neurological function, improved activities of daily living, favorable functional status, changes in cognitive functions, all-cause mortality, recurrent stroke events, and adherence to NC treatment. In addition, we will exploratively detect the biological outcomes by using the RayBiotech human inflammatory factor antibody array Q3 (RayBiotech, Inc., QAH-INF-3-1) to profile the variations of the inflammatory indicators in the blood samples. The detailed study outcomes of the VENUS registry are illustrated in Table 1.

**TABLE 1:** Complete outcomes of the VENUS registry.

Primary outcome
Distribution of scores on mRS at 12-week follow-up
Secondary outcome
Change in the NIHSS score between baseline and 4-week follow-up
Recurrent stroke events (ischemic stroke, hemorrhagic stroke which includes intracerebral hemorrhage and subarachnoid hemorrhage) within 2-week, 4-week, 8-week, 12-week, and 180-day follow-up
Composite end points of recurrent stroke events and death within 2-week, 4-week, 8-week, 12-week, and 180-day follow-up
Proportion of patients with an mRS score ≤2 at 2-week, 4-week, 8-week, 12-week, and 180-day follow-up
Distribution of scores on mRS at 2-week, 4-week, 8-week, and 180-day follow-up
Proportion of patients with the BI score ≥90 at 2-week, 4-week, 8-week, 12-week, and 180-day follow-up
Change in the NIHSS score between baseline and 2-week, baseline and 8-week, baseline and 12-week, baseline and 180-day follow-up
Proportion of patients with the BI score ≥90 at 4-week and 180-day follow-up
NC treatment duration within 2-week, 4-week, 8-week, 12-week, and 180-day follow-up
MMSE scale score at 2-week, 4-week, 8-week, 12-week, and 180-day follow up
All-cause mortality within 12-week and 180-day follow-up
Incidence of adverse events during the whole study
Exploratory outcome
^a^Variation of inflammatory indicators between baseline and 4-week follow-up

Abbreviations: mRS, modified Rankin scale; NIHSS, National Institute of Health Stroke Scale; BI, Barthel Index; MMSE, Mini-Mental State Examination; NC, Naoshuantong capsules.

a The inflammatory indicators include BLC, Eotaxin-1, Eotaxin-2, GCSF, I-309, ICAM-1, IFN-γ, IF-1 α, IL-1ra, IL-2, IL-4, IL-5, IL-6R, IL-7, IL-8, IL-10, IL-12 p40, IL-12 p70, IL-13, IL-15, IL-17A, MCP-1, M-CSF, MIG, MIP-1 β, MIP-1 δ, PDGF-BB, RANTES, TIMP-2, TNF-α, TNF-β, and TNF RI.

### Study sites and data source

**FIGURE 2:**
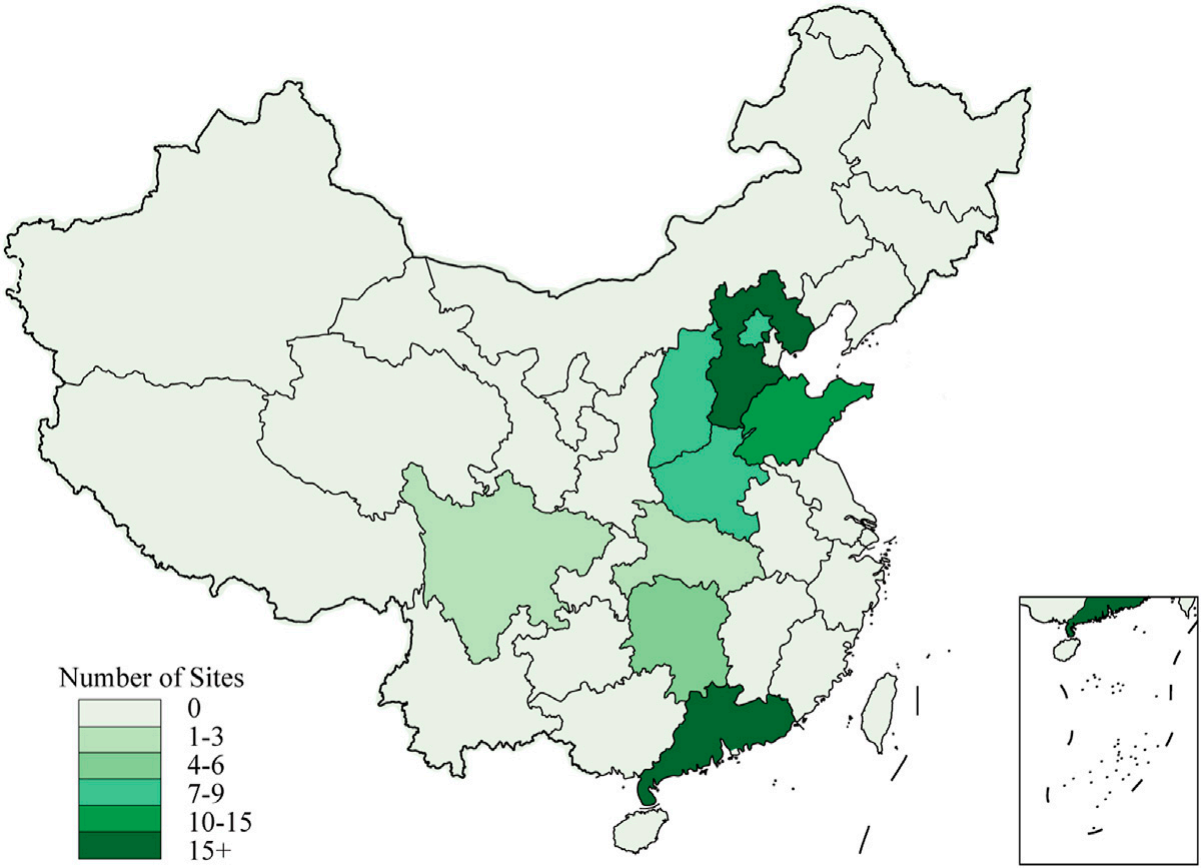
Sites distribution of the VENUS registry.

The VENUS registry steering committee will screen the ranks of hospitals in different regions of the Chinese mainland, and then evaluates their research capability and commitment to the registry with preliminary surveys. After balancing the geographical representation and research capability of the hospitals, only those hospitals that are equipped with MRI or CT machines, having experience in multicenter studies, and that can totally grasp research requirements are finally selected as participating sites of the VENUS registry. The geographical distribution of the selected 84 participating sites is shown in Figure 2.

### Quality control and management

All research personnel who participate in this study receive specific training in accordance with the protocol, processes, and details of the VENUS registry. An independent contract research organization will check for the completeness and accuracy of internal logic and the medical reference range of the PRF during the study. Any correction made on the PRF will be left with a signature and accurate date. Once the PRF is audited and approved, two research personnel will conduct back-to-back data entries into an electronic data capture system which can automatically check the completeness, accuracy, and consistency of the double-entry data, and then warn the researcher if any error or query exists. Afterward, the research personnel will check the original data and then gives feedback.

### Study sample size

Similar to other large-scale, high-quality registry studies, formal hypotheses for sample size estimation are deficient in the VENUS registry (Lei et al., 2020; Gardner et al., 2021). As a general rule, the sample size of the registry is supposed to be sufficient enough to represent the general population. Based on the crude incidence of IS in China, we planned to register 5,000 patients with IS in the VENUS registry.

### Statistical analysis

All data will be summarized as means and standard deviations for normally distributed continuous variables, while the median and interquartile range will be calculated for non-normally distributed continuous variables, and will be presented as counts and percentages for categorical variables. Comparisons will be made using parametric tests [one-way analysis of variance (ANOVA) or t-test], or nonparametric tests (chi-square test, Fisher’s exact test, Kruskal–Wallis one-way ANOVA on ranks, or Mann–Whitney *U* test) as appropriate. Multivariable regressions will be conducted with adjustments for potential covariates and the propensity score matching method will be used to minimize the influence of the other potentially unbalanced variables. Time-to-event outcomes will be presented using Kaplan–Meier methods and hazard ratios and the corresponding 95% confidence intervals will be derived from the Cox proportional hazards regression models, and the treatment effect will be assessed using the log-rank test.

## Result

**FIGURE 3:**
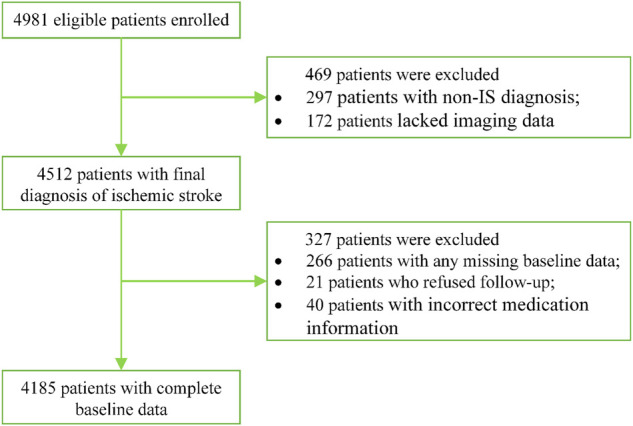
Patient enrollment flowchart.

The study is currently ongoing, and the data are continuing to be collected. A total of 4,981 eligible patients with IS have already been enrolled from April 2019 to July 2021 in 84 participating sites. There are currently 4,185 patients entering into statistical analysis after excluding 266 patients with missing baseline information, 297 patients with non-IS diagnosis, 172 patients who lacked imaging data collected upon stroke onset, 21 patients who refused follow-up, and 40 patients with incorrect medication information. The detailed patient enrollment flow chart is shown in Figure 3.

Patient demographic characteristics are presented in Table 2. Overall, 37.06% of patients were female. The average age at IS onset was 65.22 years. As for the risk factor distribution among the patients, 21.34% were current smokers, and the proportions of overweight or obesity were 42.01% and 8.75%, respectively. Hypertension was the most frequent complication and occurred in 62.37% of patients. And 24.01% of patients had diabetes mellitus, 14.19% had coronary heart diseases, and 8.77% had dyslipidemia. The proportion of patients whose course of IS was less than 14 days accounted for 93.45%, and the IS course ranged from 15 to 30 days which accounted for 6.55%. With regard to the distribution of TOAST classification, more than half of the patients had large artery atherosclerotic strokes, 34.43% had small artery occlusions, 1.98% had cardioembolic strokes, and 8.63% had stroke of the undetermined cause or other causes. Patients with an NIHSS score less than or equal to 3 accounted for 51.62% of all IS cases. In terms of the distribution of syndrome elements in IS, the internal-wind element accounted for the majority, while the yin-deficiency syndrome element, which frequently emerges during recovery or sequelae period of stroke, accounted for the smallest proportion.

**TABLE 2:** Baseline characteristics of the registered patients in the VENUS study.

Characteristic	(N = 4,185)
Age (yr), mean ± SD	65.22 ± 11.12
Gender, *N* (%)	
Male	2,634 (62.94)
Female	1,551 (37.06)
Ethnicity, *N* (%)	
Han	4,152 (99.21)
non-Han	33 (0.79)
BMI, *N* (%)	
Lean (≤18.4 kg/m^2^)	114 (2.72)
Normal (18.5–23.9 kg/m^2^)	1,947 (46.52)
Overweight (24–27.9 kg/m^2^)	1,758 (42.01)
Obese (≥28 kg/m^2^)	366 (8.75)
Education, *N* (%)	
Elementary or below	2,037 (48.67)
Middle school or high school	1,864 (44.54)
College degree	220 (5.26)
Undergraduate or above	64 (1.53)
Types of health insurance, *N* (%)	
Basic health insurance scheme	2,473 (59.09)
New cooperative medical system	1,526 (36.46)
Public medical insurance	36 (0.86)
Commercial insurance	12 (0.29)
Self-payment	138 (3.30)
Course of ischemic stroke, *N* (%)	
≤14 days	3,911 (93.45)
15–30 days	274 (6.55)
TOAST classification, *N* (%)	
Large artery atherosclerosis	2,300 (54.96)
Cardioembolism	83 (1.98)
Small artery occlusion	1,441 (34.43)
Stroke of other determined etiology	66 (1.58)
Stroke of undetermined etiology	295 (7.05)
Past medical history, *N* (%)	
Hypertension	2,610 (62.37)
Diabetes mellitus	1,005 (24.01)
Coronary heart diseases	594 (14.19)
Hyperlipidemia	367 (8.77)
Smoking status, *N* (%)	
Never smoked	2,815 (67.26)
Former smoker	451 (10.78)
Current smoker	893 (21.34)
Passive smoking	26 (0.62)
Sleep apnea syndrome, *N* (%)	90 (2.15)
Hyperhomocysteinemia, *N* (%)	564 (13.48)
NIHSS score	3.00 (2.00, 6.00)
NIHSS 0–3, *N* (%)	2,160 (51.62)
NIHSS 4–5, *N* (%)	839 (20.05)
NIHSS 6–18, N (%)	1,114 (26.62)
NIHSS >19, N (%)	72 (1.72)
mRS score	2.0 (1.0–3.0)
mRS ≤1, *N* (%)	1,754 (41.91)
1 < mRS ≤2, *N* (%)	1,152 (27.53)
3 ≤ mRS ≤5, *N* (%)	1,279 (30.56)
MMSE score	24.00 ± 7.08
BI score	79.16 ± 24.44
BI ≤ 90, *N* (%)	2,377 (56.80)
BI 90–100, *N* (%)	1,808 (43.20)
Syndrome elements in ischemic stroke, *N* (%)	
Internal-wind	2,940 (70.25)
Internal-fire	1,189 (28.41)
Phlegm-damp	1,291 (30.85)
Blood-stasis	1,182 (28.24)
Qi-deficiency	1,767 (42.22)
Yin-deficiency	756 (18.06)

Abbreviations: BMI, body mass index; OCSP, Oxfordshire community stroke project; TOAST, National Institute of Health Stroke Scale; NIHSS, National Institute of Health Stroke Scale; mRS, modified Rankin Scale; BI, Barthel Index; MMSE, Mini-Mental State Examination; NC, Naoshuantong capsules.

## Discussion

The current evidence regarding the clinical management of IS with NC has been mainly derived from RCTs. The strict selection criteria of participants within these trials limit the generalizability of the results regarding NC effectiveness in real-world practice. Generating evidence with high internal validity that is adaptive to routine clinical practice is necessary. A well-designed, large-scale registry of patients with IS merits strong attention from clinicians and clinical investigators, due to its emphasis on the association of effectiveness, benefits, and disadvantages of investigated treatment methods with long-term clinical outcomes in the real-world setting (Kim et al., 2014). In this study, the rationale and design of a prospective, multicenter, registry-based observational study with an unstrict selection criteria for target patients that aims to generate sociodemographic and medical data regarding the use of NC treatment in patients with IS are described. The benefit–harm profile of NC for patients with IS can be determined using this registry and included in updated recommended treatment guidelines.

NC is an oral Chinese medicine, and the proper use of Chinese medicine is based on the unique theory system, namely, holism and syndrome differentiation. The core concept of Chinese medicine syndrome differentiation lies in individualized treatment (Yuan, 2021). Discovering the crucial factors influencing clinical outcomes through complex and diversified information and delivering targeted medicine are important to balance the benefits and harms of specific treatments, and to maximize the net clinical benefit, achieving individualized treatment with Chinese medicine. Multidimensional data regarding NC treatment for different clinical characteristics of patients with IS in view of the Chinese medicine theory will be collected, and the corresponding effects on clinical prognosis will be explored in this registry. These results will help advance the accurate treatment of IS with NC in the theoretical system of Chinese medicine.

The global spread of the COVID-19 outbreak has presented huge challenges for public health and for conducting clinical research in terms of patient compliance and follow-up; therefore, patient enrollment is slightly delayed when compared to the anticipated completion time to ensure continuity, completeness, and accuracy of the data. As such, 4,981 patients have been enrolled in the study, of which 4,185 have been included in the final analysis. The study profile and baseline characteristics of the patients were reported. According to the current data analyses, VENUS and CNSR-III have a similar severity distribution of neurological deficits evaluated by NIHSS (Wang et al., 2019). Compared to previous national stroke registries, the VENUS registry is exclusive as it allows for the evaluation of evolution of syndrome elements at different time points of IS, which contributes to targeted, personalized precision therapy for IS based on syndrome differentiation. Moreover, the VENUS registry is closer to clinical practice in the real-world setting than rigorous RCTs. According to the current statistical analysis of baseline information, the internal-wind element comprises the largest proportion, which could be rationally explained by 93.73% of patients in the acute phase of IS and is consistent with the findings of previous studies (Xin et al., 2016). Similar to other observational registry studies, selective biases in the VENUS registry are unavoidable although the targeted sample population covers a relatively larger number of regions in the Chinese mainland (Ohman et al., 2006; Venketasubramanian et al., 2015). However, cross-sectional and longitudinal data from the multicenter participation and regular sequential follow-ups of registered patients are planned, and both the geographical and temporal characteristics of patients with IS can be explored to improve the representation of the study findings. Furthermore, special populations (such as elderly patients or patients with severe comorbidities) who are normally excluded from clinical trials will be registered based on the unstrict inclusion criteria of this registry, increasing the generalizability of the results in real-world settings. Strict quality control in terms of research personnel training and timely audits will be conducted before study initiation to ensure the accuracy and completeness of the collected data. In addition, the VENUS registry will support the establishment of a database for further individual patient data pooling as per homogenous investigational variables with that of the China Stroke Registry for Patients with Traditional Chinese Medicine (CASES-TCM) study, embedding comparative effectiveness research, pragmatic randomized clinical trial, or nested case–control study to generate secondary hypotheses for extra validation in clinical trials (Feng et al., 2021).

The VENUS registry is designed as a comprehensive, prospective, multicenter, post-marketing, observational study, aiming at evaluating the effectiveness and long-term safety of NC for patients with IS in a real-world setting. The findings of the VENUS registry will provide valuable insights into the clinical management of patients with IS and the subsequent outcomes of the uses of NC when included in the best clinical practice recommendations, to maximize the net clinical benefit of NC treatment for patients with IS.

## Abbreviations

VENUS, Vital real-world Experience regarding Naoshuantong capsules for Unselected ischemic Stroke registry; NC, Naoshuantong capsules; IS, ischemic stroke; RCT, randomized controlled trial; PRF, paper-based registry forms; OCSP, Oxfordshire community stroke project; TOAST, Trial of Org 10172 in Acute Stroke Treatment; mRS, modified Rankin scale; NIHSS, National Institutes of Health Stroke Scale; BI, Barthel index; MMSE, Mini-Mental State Examination; DSSEIS, diagnostic scale of syndrome elements in ischemic stroke; MRI, magnetic resonance imaging; CT, computed tomography; ANOVA, analysis of variance; CASES-TCM, China stroke registry for patients with traditional Chinese medicine study.
