# Supplementary material for: Vital real-world experience regarding Naoshuantong capsules for unselected ischemic stroke (VENUS): Rationale, design, and baseline of a prospective, multicenter, observational study
Source: Front Pharmacol. 2022 Oct 5;13:933258. doi: 10.3389/fphar.2022.933258 (PMC9579274; doi:10.3389/fphar.2022.933258)
Supplement: Supplementary file 1 [file DataSheet1.pdf]

Supplementary Materials

Appendix 1. Standard formulation of naoshuantong capsules.

Appendix 2. High performance liquid chromatograph of naoshuantong capsules.

Appendix 1. Standard formulation of naoshuantong capsules.

Pinyin name	Complete species name	Authorities / family name	Scientific name	Proportion (g)
Pu Huang	Typha angustifolia L.	Typhaceae	Typhae Pollen	0.890
Chi Shao	Paeonia lactiflora Pall.	Paeoniaceae	Paeoniae Radix Rubra	0.635
Yu Jin	Curcuma longa L.	Zingiberaceae	Curcumae Radix	0.510
Tian Ma	Gastrodia elata Blume	Orchidaceae	Gastrodiae Rhizoma	0.255
Lou Lu	Leuzea uniflora (L.) Holub	Asteraceae	Rhapontici Radix	0.380

Appendix 2. High performance liquid chromatograph of naoshuantong capsules.

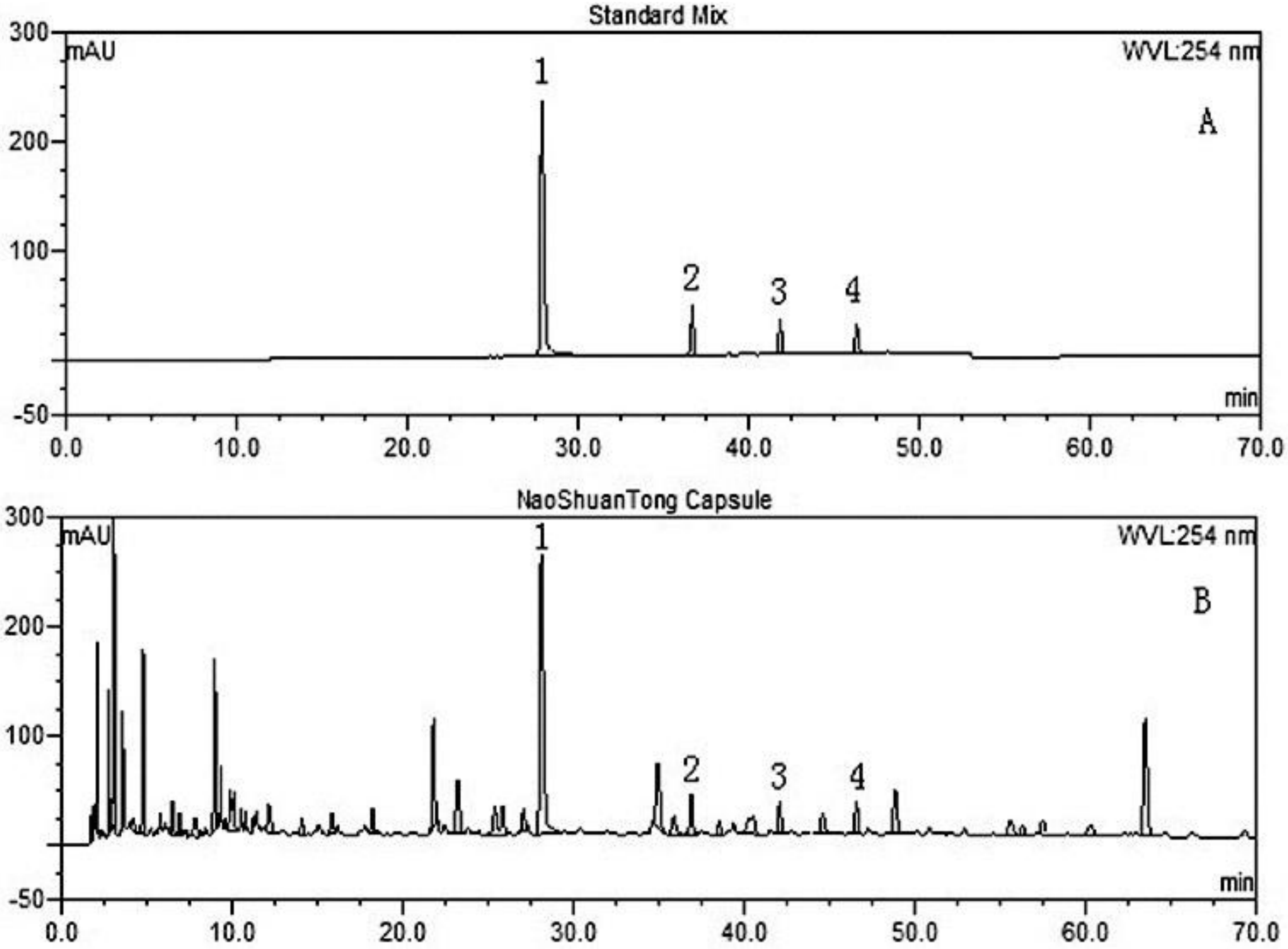

High performance liquid chromatograph of standard mix and naoshuantong capsules using ultraviolet absorbance detection at wavelength 254 nm.

1: Paeoniflorin; 2: Ecdysterone; 3: Typhaneoside; 4: Isorhamnetin-3-O-neohesperidoside.

Reference:

Liu H, Peng YY, Liang FY, et al. Protective effects of traditional Chinese medicine formula NaoShuanTong capsule on haemorheology and cerebral energy metabolism disorders in rats with blood stasis. Biotechnol Biotechnol Equip. 2014 Jan 2;28(1):140-146. doi: 10.1080/13102818.2014.901678.
